# Integrative medicine rehabilitation for children with cerebral palsy: a study protocol for a multicenter pragmatic randomized controlled trial

**DOI:** 10.1186/s13063-020-04639-x

**Published:** 2020-08-17

**Authors:** Mi-Joo Lee, Young-Ju Yun, Sun-ae Yu, Yong-Beom Shin, Soo-Yeon Kim, Jun-hee Han

**Affiliations:** 1 Department of Korean Gynecology, CHA Bundang Medical Center, CHA University, Seongnam-si, Gyeonggi-Do South Korea; 2 Department of Integrative Medicine, School of Korean Medicine, Pusan National University, 20, Geumo-ro Mulgeum-eup, Yangsan-si Gyeongsangnam-do, 50612 Pusan, Republic of Korea; 3 Department of Pediatrics, College of Korean Medicine, Dong-Eui University, Pusan, South Korea; 4 Department of Rehabilitation Medicine, Pusan National University School of Medicine and Biomedical Research Institute, Pusan National University Hospital, Pusan, South Korea; 5 Department of Rehabilitation Medicine, Pusan National University Yangsan Hospital, Pusan National University School of Medicine, Yangsan, South Korea; 6 Department of Statistics & Institute of Statistics, Hallym University, Chuncheon, South Korea

**Keywords:** Cerebral palsy, Rehabilitation, Gross motor function measure (GMFM), Integrative medicine rehabilitation (IMR), Goal attainment scale (GAS), Korean Bayley scales of infant development III (K-BSID III), Pediatric quality of life inventory (PedsQL), Children, Randomized controlled trial

## Abstract

**Background:**

Traditional Korean medicine (TKM) has been employed for the treatment of children with cerebral palsy in Korea; however, the addition of TKM to usual rehabilitation (UR) treatment is hindered by insufficient evidence of clinical improvement with TKM in patients with cerebral palsy. In this study, we will evaluate the effectiveness and safety of integrative medicine rehabilitation (IMR) for cerebral palsy through a randomized controlled clinical study.

**Methods:**

Eighty children (2–6 years old) diagnosed with cerebral palsy will be recruited and randomly divided into groups A and B. Patients in group A will receive IMR with UR, while those in group B will receive only UR during weeks 1–12 of the study. IMR includes acupuncture treatment (head and limb acupuncture) three times a week and the administration of herbal medicine (Yukgunza-tang and Yukmijihwang-tang extracts) twice a day in parallel with UR. Evaluations will be conducted at the beginning of the study and at 12 and 24 weeks (follow-up). The primary outcome is the Gross Motor Function Measure-88 score, and the secondary outcomes are the scores for the Goal Attainment Scale, Korean Bayley Scales of Infant Development III, and the Pediatric Quality of Life Inventory, and adverse events.

**Discussion:**

This will be the first pragmatic randomized controlled trial to evaluate the efficacy and safety of IMR in children with cerebral palsy in Korea. The results will help to demonstrate if IMR is an effective therapeutic approach for cerebral palsy.

**Trial registration:**

Ministry of Food and Drug Safety 31361 (http://www.mfds.go.kr). Registered on 29 June 2017. Clinical Research Information Service KCT0002620 (https://cris.nih.go.kr/cris/search/search_result_st01.jsp?seq=9819). Registered on 29 December 2017.

## Background

The worldwide prevalence of cerebral palsy (CP) is estimated to be 2–2.5 cases per approximately 1000 survivors, with a prevalence rate of 0.26% among children in Korea. Korea has the lowest fertility rate among the Organization for Economic Cooperation and Development countries. However, with the increase in the number of multiple births owing to the increase in in vitro fertilization and the subsequent increase in the proportion of preterm infants, the prevalence of CP is increasing [1].

Children with CP show various restrictions on physical activity due to impaired motor functions and posture. Thus, CP treatment involves multiple approaches including usual rehabilitation (UR; physiotherapy, occupational therapy, speech therapy, and orthopedic rehabilitation therapy), injection therapy, medication, and surgical treatment [2]. Traditional Korean medicine (TKM) has also been employed for the rehabilitation of children with CP [3–5]. Integrative medicine rehabilitation (IMR) is the addition of acupuncture and herbal medicine treatment to UR for CP. Numerous clinical studies in China have reported the remarkable efficacy as well as the adverse effects of acupuncture treatment, chuna therapy, and herbal medicine treatment in children with CP [6]. However, the effectiveness of IMR in patients with CP is still controversial in Korea as the Korean and Chinese traditional medicine theories and clinical applications are different which restrict extrapolation. More definitive clinical studies are needed for the medical insurance coverage of TKM treatment for CP.

### Objectives

To address this lacuna in the literature, this study aims to administer acupuncture and herbal medicine treatment simultaneously with UR therapy for 12 weeks and compare the findings with those obtained using UR alone. The Gross Motor Function Measure-88 (GMFM-88), Goal Attainment Scale (GAS), Korean Bayley Scales of Infant Development III (K-BSID-III), and Pediatric Quality of Life Inventory (PedsQL) scores have been selected as outcome variables. A rigorously designed large-scale multicenter pragmatic randomized trial will clarify whether IMR can facilitate clinical improvement in children with CP.

### Trial design

Multicenter, pragmatic, randomized-controlled, evaluator-blinded trial.

## Methods/design

### Patients

#### Inclusion criteria

Participants must meet the following conditions to be eligible: (1) aged 2–6 years and diagnosed with CP according to a combination of clinical findings and cerebral magnetic resonance imaging and (2) parental consent for participation in the study.

#### Exclusion criteria

Participants meeting any of the following criteria will be excluded: (1) orthopedic surgery in the preceding 6 months; (2) Botox treatment in the preceding 6 months; (3) a history of degenerative brain disease, congenital neuromuscular disease, or uncontrolled seizures; (4) participation in other clinical trials in the preceding 3 months; (5) a history of serious diseases (such as tumors, heart disease, or severe infectious diseases) other than CP; (6) difficulty accepting acupuncture and/or herbal medicine treatment; (7) abnormalities in the degradation and absorption of lactose or galactose; (8) a history of liver- or kidney-related diseases; and (9) sensitivity to medicines.

### Patient and public involvement

Although not directly involving patients, the number and duration of acupuncture treatments and patient position for acupuncture administration were determined for the study design to ensure patient comfort in consultation with a skilled TKM doctor and physical therapist experienced with treating CP patients.

The individual goals for GAS (secondary outcome measure) will be set by the patients, guardians, and the researcher; however, they will be assessed by the researchers only. The guardians submitting the written consent will also complete the PedsQL™ questionnaire for the quality of life evaluation of children with CP. The results will be disseminated by publication in a peer-reviewed journal, presentation at international congresses, and via telephonic communication to the patients.

### Study design

This is a multicenter pragmatic randomized controlled evaluator-blinded clinical trial of the effectiveness of IMR for CP. We will recruit participants by advertising on the Internet and through posters in hospitals and communities. The recruiting period will be from January 1, 2021, to December 31, 2022.

After explaining the “Guide for Study Subjects” to the participants’ guardian, their consent for clinical study participation will be obtained by the study investigators. The Guide for Study Subjects contains information on the effects of the study and adverse reactions as well as the safety data. The evaluations will be carried out after receipt of the consent forms. On the consent form, participants will be asked if they agree to the use of their data should they choose to withdraw from the trial. Participants will also be asked for permission for the research team to share relevant data with people from the Universities and hospitals taking part in the research. This trial does not involve collecting biological specimens for storage.

The participants will be randomly stratified into groups A and B. Randomization will be performed based on the Gross Motor Function Classification System (GMFCS) stage (stages 1–3 vs. stages 4–5), age (1–2 years vs. 4–6 years), and CP type (spastic vs. non-spastic). The principal researcher will use a block randomization method using a random block size to generate the random allocation sequence. Computer-assisted randomization will be performed using the Excel 2010 program, and the randomized sequences will be delivered in an opaque envelope to conceal allocation. After screening, the researcher will open the sealed envelope in the presence of the participant, and the assignment result will be confirmed.

**Fig. 1:**
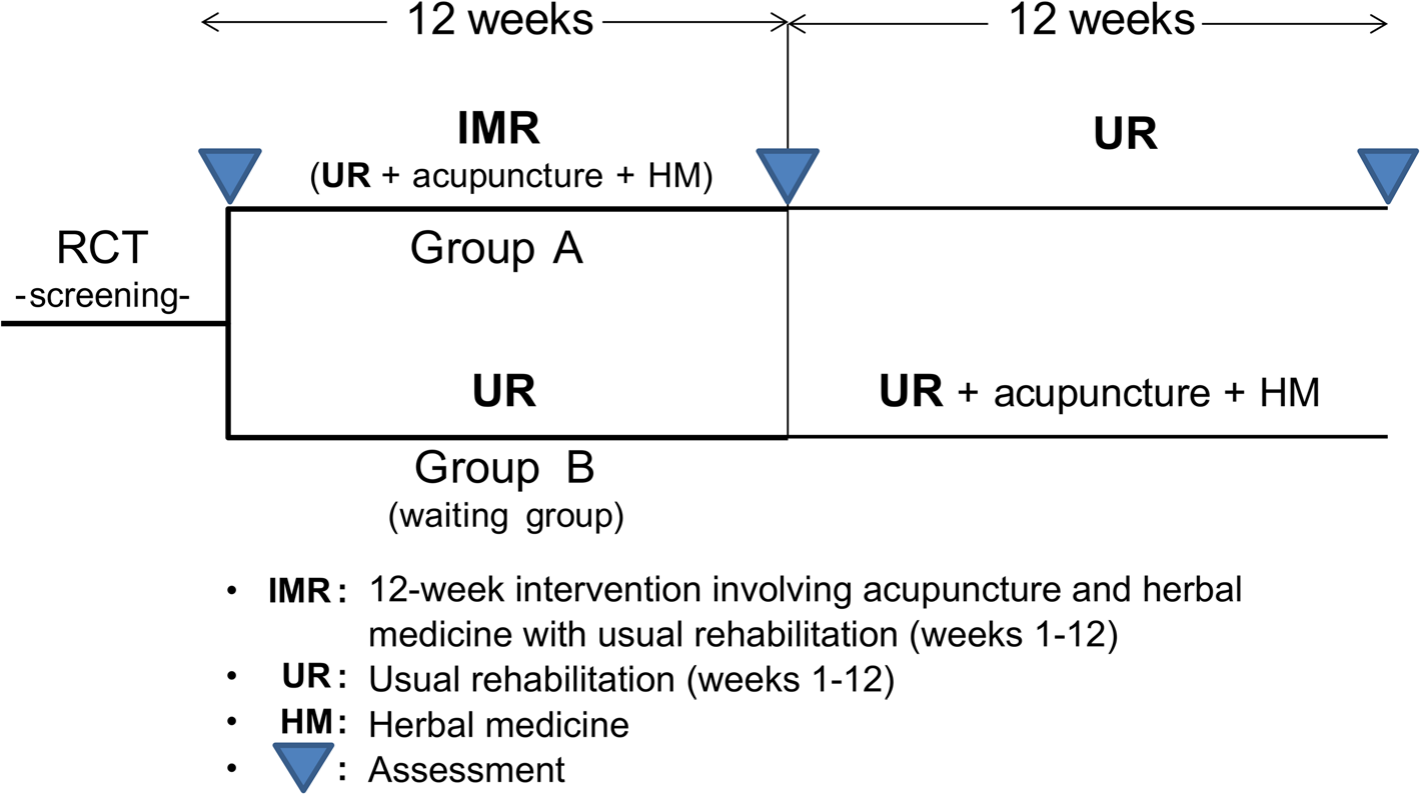
Flow chart of the study design. First, we will recruit 80 participants from multiple centers. Participants will be randomly assigned to groups A (*n* = 40) and B (*n* = 40) after baseline assessment. Second, all participants will continue UR (physiotherapy, occupational therapy, etc.), as they had been doing previously, throughout the 24-week study period. Third, over weeks 1–12, group A will receive additional IMR comprising acupuncture and herbal medication with UR, and group B is a waiting group that will receive only UR. Participants will be assessed at 12 weeks. Fourth, group B will receive 12 weeks of acupuncture and herbal medication with UR during weeks 13–24; group A will continue UR during weeks 13–24 and return to the institution for follow-up assessment at 24 weeks. The researchers will then collect the results and analyze the data. Abbreviations: UR, usual rehabilitation; IMR, integrative medicine rehabilitation

**Fig. 2:**
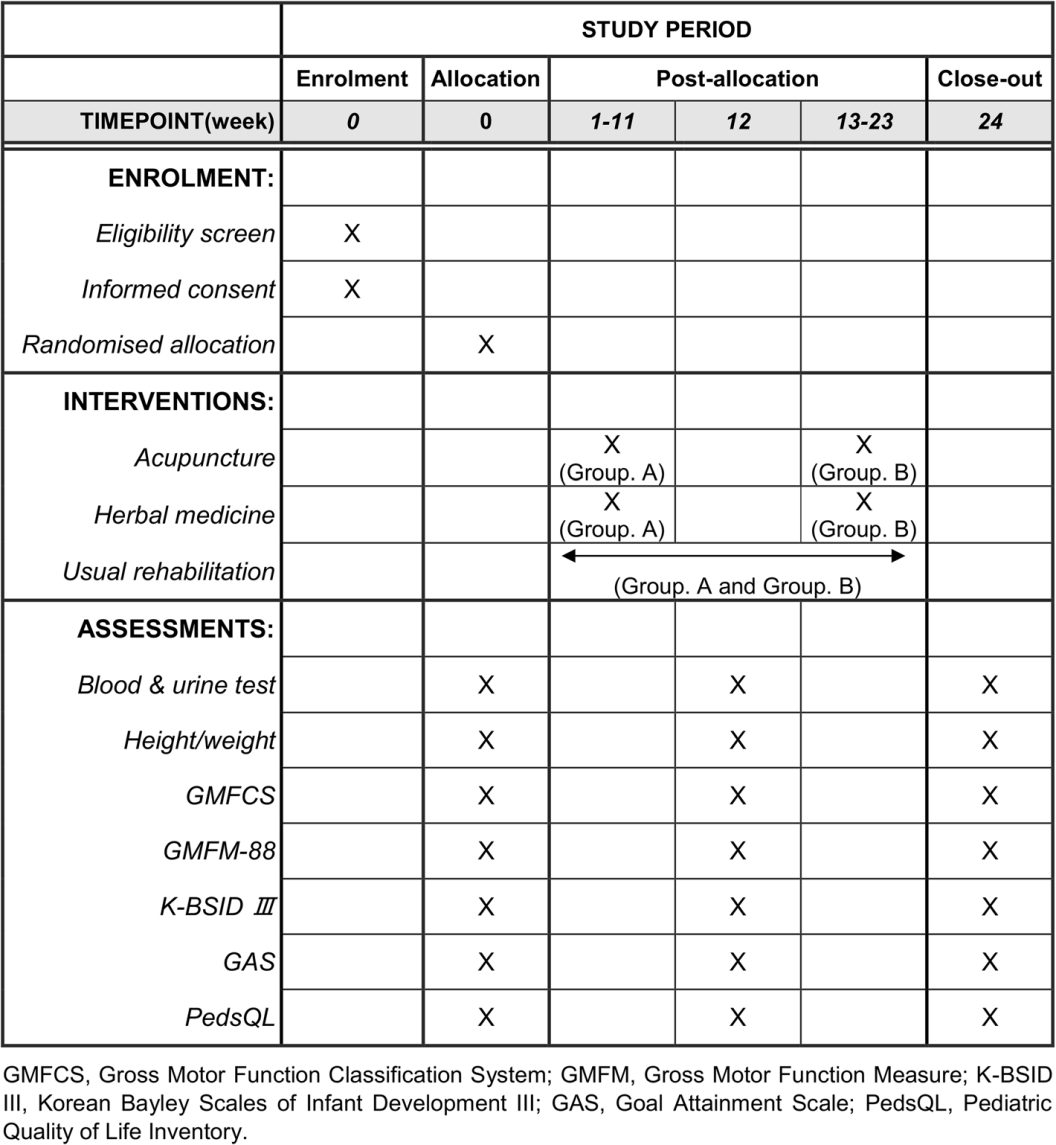
Standard Protocol Items: Recommendation for Interventional Trials (SPIRIT) schedule of enrollment, interventions, and assessments. GMFCS, Gross Motor Function Classification System; GMFM, Gross Motor Function Measure; K-BSID III, Korean Bayley Scales of Infant Development III; GAS, Goal Attainment Scale; PedsQL, Pediatric Quality of Life Inventory

This is a pragmatic clinical trial; hence, all the participants will continue their UR (physiotherapy, occupational therapy, etc.) throughout the 24-week study period. The type of UR will be unrestricted; the only requirement is that it needs to be conducted 4–5 times a week, as children with CP in Korea receive UR 5.74 times per week on average [7]. Group A will additionally receive IMR comprising acupuncture and herbal medication with UR for 12 consecutive weeks. Group B is a waiting group that will receive only the UR without acupuncture and herbal medicine for the initial 12 weeks. However, to comply with the standard of care for CP in Korea, group B will receive additional 12 weeks of IMR as their only formal therapy from weeks 13–24; group A will return to the institution for assessment at 24 weeks (Figs. 1 and 2).

The assessments will be performed three times in total: at baseline, 12 weeks (after 3 months), and 24 weeks (after 6 months). Two physical therapists with more than 5 years of experience in pediatric rehabilitation will evaluate the scores for each item and the recorded videos (the videos will be recorded by a physical therapist during assessment to evaluate the participants’ GMFM-88, GAS, and K-BSID III developmental status). In principle, the same person should conduct all three evaluations. Assessors will be blinded to the subject assignment, and an inter-rater reliability test will be conducted before each study. The proposed study protocol is schematically presented in Figs. 1 and 2.

Sample size calculations are based on a previous study [8]. We estimated a GMFM-88 score difference of 2.8 ± 3.2 (effect size, *d* = 0.8) which is considered to clinically indicate a change in motor function. A two-sided 5% significance level and 90% power were considered, and calculations indicated the requirement of approximately 28 participants in each group. Estimating a 30% dropout rate, each group will require 40 initial participants. Therefore, this study will be performed as a multicenter randomized controlled trial (RCT) by recruiting 80 subjects for a period of 2 years. Patients will be recruited from Pusan National University Hospital, Yangsan Pusan National University Hospital, Pusan Dongeui Hospital, and Bundang Cha Hospital.

In the event of a direct injury in relation to this study, appropriate medical action may be taken according to the decision of the clinical researcher. The level of compensation shall be an appropriate amount for the nature of the damage, its degree of consistency, and as would be generally decided by the courts in Korea and cover the entire treatment cost until the adverse event (AE) is cured. If rehabilitation therapy is required or an after-effect is noted, appropriate compensation will be provided after assessing the disorder.

### Interventions

#### Acupuncture treatment

The acupuncture treatments are described in detail below and in Table 1.

**Table 1:** Standards for Reporting Interventions in Clinical Trials of Acupuncture (STRICTA) checklist for the study

Item	Detail
**1. Acupuncture rationale**	1a) Each patient will be treated with non-local needle acupuncture (according to the theory of channels of TKM) at distant points and press needles on stiff joints.
	1b) Acupuncture point selection will be based on Acupuncture Medicine.^9^
	1c) Acupuncture points are based on the general principles of TKM; however, the treatment will be modified over the course of the study to accommodate the individual’s changing pattern of pain, edema, or other health issues.
**2. Details of needling**	2a) The protocol will allow for up to 17 needles and eight press needles per treatment. The number of acupuncture sessions will be modified according to the judgment of the practitioners within the maximum allowance.
	2b) The acupuncture points consist of LI4 (HeGu), LI11 (QuChi), LU9 (Tai Yuan), LR3 (Taichong), ST36 (ZuSanLi), and BL60 (KunLun) on both extremities and GV20 (Bai Hui) and EX-HN-1 (Si Shen Cong) on the head. The press needle acupuncture points consist of LI5 (Yangxi) and TE4 (Yangchi) for upper limb paralysis (affected side) and BL60 (Kunlun) and KI3 (Taisi) for lower limb paralysis (affected side) in spastic palsy. In case of adverse reactions such as hematoma or pain, the treatment at that point will be skipped.
	2c) The depth of needle insertion varies with the thickness of the skin and subcutaneous fatty tissues at the acupuncture points; a depth of 1–5 mm is usually recommended.
	2d) The practitioner will determine the appropriate stimuli based on the patient’s reaction by assessing changes in palpatory findings instead of employing vigorous manipulation.
	2e) Manual stimulation will be provided via the acupuncture needles without using twisting or twirling manipulation.
	2f) Body acupuncture will be retained for up to 20 min or withdrawn immediately after the insertion of needles based on the patient’s reaction. The practitioner will provide directions for the removal of the press needle before washing hands and feet after returning home.
	2 g) The practitioner will use 0.2 mm × 1.5 mm sterilized stainless-steel needles (DONGBANG Acupuncture Inc., Korea) for body acupuncture and 0.18 mm × 1.3 mm × 1.5 mm (DONGBANG Acupuncture Inc., Korea) press needles. TKM doctors will conform to the Clean Needle Technique so as to avoid hand contamination.
**3. Treatment regimen**	3a) Group A will undergo acupuncture treatment three times a week over weeks 1–12. Group B will undergo acupuncture treatment three times a week over weeks 13–24. Each group will receive a total of 36 acupuncture treatments.
	3b) Acupuncture treatment will be administered a maximum of three times every week in 20-min sessions.
**4. Other components of treatment**	4a) For patients with spastic palsy, the practitioner will allow the patient to exercise the joint for 1 min after applying the press needle. 2. In addition to needling, thermal stimulation of the acupuncture points will be performed. 3. The patients will perform self-management activities (exercise therapy, etc.) 4. The patients will also receive herbal medicine three times per day over a period of 12 weeks parallel to the acupuncture treatment.
	4b) Patients will be informed about acupuncture in the study as follows: “In this study, a TKM doctor with more than 5 years of clinical experience will safely conduct the procedure and use pediatric disposable acupuncture needles.”
**5. Practitioner background**	5) A TKM doctor with more than 5 years of clinical experience.
**6. Control or comparator interventions**	6a) Restricting the type and frequency of rehabilitation treatments limits the potential benefits of rehabilitation therapy and is not consistent with research ethics. According to a prior observational study, children with CP in Korea undergo UR 5.74 times per week on an average [7]. Based on this, the minimum number of rehabilitation treatments is set at twice a week
	6b) The control group will continue to receive UR at least twice a week during the study period (types and number of rehabilitation treatments and rehabilitation institutions for UR are not limited).

*UR* usual rehabilitation, *TKM* traditional Korean medicine

#### Body acupuncture (0.2 mm × 1.5 mm, DONGBANG Acupuncture Inc., Korea)

The following acupoints will be used: LI4 (HeGu), LI11 (QuChi), and LU9 (Tai Yuan) for the upper limbs (both); LR3 (Taichong), ST36 (ZuSanLi), and BL60 (KunLun) for the lower limbs; and GV20 (Bai Hui) and EX-HN-1 (Si Shen Cong) for the head. These acupoints were identified with reference to the Korean Medicine Convergence Research Information Center’s standard acupuncture database. Clinical and experimental studies on acupuncture treatment in CP have shown that LI4 (HeGu), LI11 (QuChi), and LU9 (Tai Yuan) can be used to improve upper limb stiffness and LR3 (Taichong), ST36 (ZuSanLi), and BL60 (KunLun) to improve lower limb rigidity. GV20 (Bai Hui) and EX-HN-1 (Si Shen Cong) can be used to improve cognitive function in CP [9–11].

Patients will receive acupuncture treatment in their most comfortable position (supine, lateral, or sitting position) without limiting their posture. A TKM doctor will conduct the acupuncture treatment at a depth of 1–5 mm without twisting and twirling manipulation. After 20 min, the TKM doctor will remove the needle and disinfect the area of the needle with alcohol. The patient will rest for 5 min after the acupuncture treatment.

#### Press needle (0.18 mm × 1.3 mm × 1.5 mm, DONGBANG Acupuncture Inc., Korea)

Patients with spastic CP will receive press needle treatment to reduce their muscle tone at the following acupoints: LI5 (Yangxi) and TE4 (Yangchi) for upper limb paralysis (affected side) and BL60 (Kunlun) and KI3 (Taisi) for lower limb paralysis (affected side). After applying the press needle, the practitioner will allow the patient to exercise the joint for 1 min and direct the patient to remove the press needle before washing hands and feet on returning home.

#### Herbal medicine

The herbal medicine used in this study is a combination of Yukgunza-tang and Yukmijihwang-tang extracts prepared by Kracie Pharma Korea Co. which is licensed by the Korea Food and Drug Administration and produced by a GMP-approved facility. The Yukgunza-tang extract contains the following raw materials (in an adult dose per day basis, 6 g): Ginseng Radix 4.0 g, Atractylodes Rhizoma Alba 4.0 g, Smilacis Rhizoma 4.0 g, Pinelliae Tuber 4.0 g, Fraxini Cortex 2.0 g, Zizyphi Fructus 2.0 g, Glycyrrhizae Radix 1.0 g, and Zingiberis Rhizoma 0.5 g. The Yukmijihwang-tang extract contains the following raw materials (on an adult dose per day basis, 6 g): Rehmanniae Radix 5.0 g, Cornus Fructus 3.0 g, Dioscoreae Rhizoma 3.0 g, Alismatis Rhizoma 3.0 g, Smilacis Rhizoma 3.0 g, and Moutan Cortex 3.0 g. Yukmijihwang-tang helps improve cognitive functions, self-organization, and social development [5, 12–15], and Yukgunza-tang improves gastrointestinal functions which facilitate food intake and promote growth in children [16, 17].

The medication doses are as follows: (1) for patients aged 12–47 months, a total dose of 4 g per day (Yukgunza-tang, e.g., 2 g, + Yukmijihwang-tang, e.g., 2 g), and (2) for patients aged 48–72 months, a total dose of 6 g per day (Yukgunza-tang, e.g., 3 g, + Yukmijihwang-tang, e.g., 3 g). The pediatric dose has been determined in accordance with the TKM pediatric science textbook. Patients will consume the herbal medicine with drinking water twice a day about 1 h after breakfast and lunch.

The researcher will check if the participant is taking the herbal medicine twice a day and will provide 2–3 days of herbal medicine at each visit. If the patient is unable to consume the herbal medicine, the duration of and reason for the discontinuation will be recorded on a separate chart. The researcher will check whether the stock and usage records of the herbal medicine match.

### Outcome measures

Assessment data will be collected at the study initiation and at 12 and 24 weeks thereafter (Table 2).

**Table 2:** Overview of the outcome measures at different time points in the study

Study week	Screening	Baseline	1–11 wks	12 wks	13–23 wks	24 wks
Informed consent	●					
Inclusion/exclusion criteria	●					
Demographics	●					
Concomitant disorders	●					
Medical history	●					
Medication	●					
Adverse events/vital signs		●	●	●	●	●
**Assessment**						
Blood and urine test		●				●
Height/weight		●		●		●
GMFCS		●		●		●
GMFM-88		●		●		●
K-BSID III		●		●		●
GAS		●		●		●
PedsQL		●		●		●

*wks* weeks, *GMFCS* Gross Motor Function Classification System, *GMFM* Gross Motor Function Measure, *K-BSID III* Korean Bayley Scales of Infant Development III, *GAS* Goal Attainment Scale, *PedsQL* Pediatric Quality of Life Inventory

### Baseline assessments

The population statistics data include sex and age. The researcher will examine the patient’s height, weight, complex disease history, health problems, medication, rehabilitation status, and functional food intake. Blood and urine tests will be performed to check for liver- or kidney-related diseases. The blood test will include assessments for aspartate aminotransferase, alanine transaminase, total bilirubin, blood urea nitrogen, and creatinine. The urine test will include assessments of pH, protein and glucose levels, and occult blood. Prior to random selection, the patient’s GMFCS score will be evaluated by physical therapists who have completed the evaluation tool training.

### Primary outcome measurement

#### GMFM-88

The GMFM-88 is a standardized observational instrument to measure changes in gross motor function over time in children with CP. The GMFM-88 has five dimensions: A—lying and rolling (17 items); B—sitting (20 items); C—kneeling and crawling (14 items); D—standing (13 items); and E—walking, running, and jumping (24 items). The items are scored from 0 to 3 (0 = not starting, 1 = starting to move but performing within 10% of functional activity, 2 = performing from 10% to less than 100% of functional activity, 3 = performing functional activity perfectly). The GMFM-88 total score is calculated as the mean score of all five dimensions, and the goal total score is the mean of the scores for individual dimensions selected by assessors considering the GMFCS and age [18, 19]. In this study, changes in the total score and goal total scores at baseline, 12 weeks, and 24 weeks will be used as evaluation variables.

### Secondary outcome measures

#### GAS

The GAS developed by Kiresuk allows the establishment of appropriate and clear treatment goals and the measurement of treatment progress according to the individual goals [20]. Each goal is rated on a 5-point scale with the degree of attainment captured for each goal area: 0 = achievement of the expected level, 1 = better than expected outcome (somewhat better) or + 2 (much better), − 1 = worse than expected outcome (somewhat worse) or − 2 (much worse). GAS will be assessed by researchers.

#### K-BSID III

The K-BSID III is an individually administered instrument to measure the developmental functioning of infants and toddlers. It is appropriate for administration to children aged 1–42 months; hence, it will be used only for infants under the age of 42 months. The K-BSID III includes cognitive, language, motor, social-emotional, and adaptive behavior scales [21]. In this study, a licensed physical therapist who has undergone rigorous training in accordance with the recommendations in the K-BSID III guidelines will assess the participants in each area. Changes in the composite scores in each area at baseline and at 12 and 24 weeks will be used as evaluation variables.

#### PedsQL™

The PedsQL™ Cerebral Palsy Module is a measurement model to measure health-related quality of life (HRQOL) in children with CP [22]. The PedsQL™ Cerebral Palsy Module includes daily activities, movement and balance, pain and hurt, fatigue, eating activities, school activities, and speech and communication. The score is rated on a 5-point scale (0 = never a problem; 1 = almost never a problem; 2 = sometimes a problem; 3 = often a problem; 4 = almost always a problem). Each item will be reverse-scored and linearly transformed to a 0–100 scale (0 = 100, 1 = 75, 2 = 50, 3 = 25, and 4 = 0). Higher scores indicate better HRQOL. In this study, the Korean version of the PedsQL™ 3.0 Cerebral Palsy Module questionnaire [23] will be used, and the changes in the scores at baseline and 12 and 24 weeks will be used as evaluation variables.

### Adverse events

Information regarding AEs will be collected through patient symptom reports and observations by researchers at each visit. The type of adverse reaction, severity, and causal relationship with the treatment will be determined and recorded in the case record. The severity of AEs will be assessed using Spilker’s three-step classification: 1 = mild (treatment is not needed, and the patient’s normal functioning is not significantly inhibited), 2 = moderate (the patient’s normal functioning is significantly inhibited), and 3 = severe (a high level of treatment is required, and the patient shows after-effects).

The AEs of acupuncture may include local inflammation, discomfort, sputum hemorrhage and bruising, pruritus, fever, and other acupuncture-related risks [24]. The AEs of herbal medicine may include indigestion, diarrhea, and urticaria [25]. To evaluate the safety of herbal medicine, blood and urine tests will be conducted for all participants at baseline and 24 weeks. In case of symptoms of drug-induced liver damage during the clinical study, liver function tests will be performed immediately after the discontinuation of herbal medicines with the assessment of drug-induced hepatitis using the drug-induced liver injury causality assessment scale [26].

### Statistical analysis

Researchers will analyze for our main objective by comparing the outcomes (GMFM-88) of children receiving UR with IMR (group A) and those receiving UR without acupuncture and herbal medicine (group B) between baseline and 12 weeks. Within-group comparisons for changes in the duration of IMR and UR will be performed. We will use linear mixed models to test group differences in improvements over time for each outcome measure while adjusting for the serial correlation introduced by repeated measurements on the same participants. Group assignment, GMFCS level, time, and their interactions were included as fixed effects, with age as a fixed-effect covariate; patient and patient-by-time effects were included as random effects. A heterogeneous autoregressive covariance structure will be used for the random effects. Rates of change (slopes) from baseline to 12 weeks and from 12 to 24 weeks will be tested to evaluate treatment effects. Rates of change from baseline to 24 weeks will be compared with overall improvement across outcomes. Missing values will be imputed using the last-observation-carried-forward method.

### Data collection and management

During the recruitment period, researchers will record the baseline characteristics of participants in clinical record forms (CRFs). Data recorded on the original CRFs will be entered into Excel spreadsheets by two data managers independently. The data managers will cross-check the two data sets to ensure accuracy.

All documents related to clinical studies, such as CRFs, should be recorded and distinguished by the subject identification code, usually the subject’s initials, not the name. Records identifying the subject’s identity will be kept confidential, and the identity will also remain confidential on publication of the results of the clinical study.

If a subject prefers to discontinue participation, the researcher can stop the clinical study for the patient and arrange all the planned assessments at the final visit. In the case of a failed follow-up, researchers must demonstrate “reasonable effort” to contact the subject by documenting the actions taken (e.g., telephone contact date, registered mail, etc.) For patients opting to drop-out from the clinical trials, visits should be scheduled as soon as possible to conduct all the planned assessments at the final visit.

The datasets analyzed during the current study will be available from the corresponding author on reasonable request.

### Monitoring

If one of the following is encountered, it is possible to terminate the clinical trial early: (1) disease condition of the subject deteriorates and it is difficult to continue the research; (2) unexpected systemic diseases that were not detected before the study are diagnosed; (3) the subject or guardian requests the suspension of the examination; (4) self-treatment which may affect the judgment of the study results during the procedure period or the observation period; (5) the occurrence of serious AEs or the patient requires test termination due to AEs; (6) death of the subject; (7) violation of the plan for herbal medicines and medication; (8) subject cannot be tracked; (9) it is difficult for the research to proceed because of the medical staff’s judgment; and (10) treatment compliance is less than 50% (acupuncture or herbal medicine treatment).

Monitoring of clinical trials and auditing trial conduct will be carried out by the company designated by Pusan National University Hospital TKM Clinical Research Center via a regular once-monthly visit or telephonically. A Data Monitoring Committee was not considered as this was a low-risk intervention.

Monitoring personnel will monitor the progress of the clinical study and consult the researcher in case of a problem. Only the Principal Investigator can access the final dataset, and statisticians will be provided with a dataset blinded to group allocation. However, the TKM doctor cannot be blinded to group allocation, because acupuncture is possible only when the participant and the TKM doctor have contact.

Protocol amendments will be performed based on consensus by the Pusan National University Hospital TKM Clinical Research Center, and researchers will update the protocol in the Ministry of Food and Drug Safety Integrated Information System.

## Discussion

The aim of this trial is to investigate the efficacy and safety of IMR for children with CP in Korea. The results will help to demonstrate if IMR is an effective therapeutic approach for CP.

In a systematic review of the effects of acupuncture and herbal medicines in Chinese children with CP, six out of 35 RCTs reported AEs; however, these were not associated with acupuncture and herbal medicine treatment [6]. Despite the safety of acupuncture and herbal medicines, no large-scale RCTs have been conducted in Korea. This lack of evidence necessitates an additional RCT to evaluate the clinical efficacy of integrative medicine in patients with CP.

The acupuncture program includes body acupuncture and press needle acupuncture. The acupoints for body acupuncture include five head points (GV20, EX-HN1) and 12 limb points (bilateral LI4, LI11, LU9, ST36, LR3, and BL60). In cases of spastic CP, additional press needles will be used on the wrists and ankle joints (LI5, TE4, BL60, and KI4). A systematic review of literature in Korea and China has identified these acupoints to be effective for paralysis and cognitive impairment [10, 27, 28].

The herbal medicine to be used in this study is a combination of Yukgunza-tang extracts and Yukmijihwang-tang extracts. Yukmijihwang-tang extract has been reported to improve cognitive and motor function in experimental and clinical studies in children with CP [5, 12–15]. In contrast, Yukgunza-tang extracts have been used to improve the gastrointestinal function of children with CP, promote the absorption of Yukmijihwang-tang extract (K-13), and prevent digestive disorders as a result of Yukmijihwang-tang extract (K-13) administration [16, 17].

The primary outcome is the GMFM-88 score which assesses large motor function in CP. The secondary outcomes are the GAS, K-BSID III, and PedsQL™ scores and AEs. A limitation of this study is that UR is not fully controlled because this study is a pragmatic RCT. This study could potentially confirm whether integrative medicine is an effective rehabilitation therapy for CP. Our study may show that IMR is effective and safe for treating motor dysfunction, language disorder, and social-emotional disorders in children with CP.

### Trial status

Protocol version number and date: 20170079446_Ver 1.2 and 21 June 2017.

Date recruitment will begin: January 2021.

Approximate date when recruitment will be completed: December 2022.

## Supplementary information

**Additional file 1.** SPIRIT checklist

## Data Availability

Only the principal investigator of this study can access the final dataset and disclose contractual agreements, which limits the access for other investigators. The data generated here will be deposited at the Clinical Research Information Service (https://cris.nih.go.kr/) and other researchers can access those data upon request.

## Abbreviations

AE: Adverse event
CP: Cerebral palsy
CRFs: Clinical record forms
GAS: Goal Attainment Scale
GMFCS: Gross Motor Function Classification System
GMFM-88: Gross Motor Function Measure-88
HRQOL: Health-related quality of life
IMR: Integrative medicine rehabilitation
K-BSID III: Korean Bayley Scales of Infant Development III
PedsQL: Pediatric Quality of Life Inventory
RCT: Randomized controlled trial
TKM: Traditional Korean medicine
UR: Usual rehabilitation
